# Cannabinoid-based drugs targeting CB_1_ and TRPV1, the sympathetic nervous system, and arthritis

**DOI:** 10.1186/s13075-015-0743-x

**Published:** 2015-09-06

**Authors:** Torsten Lowin, Rainer H. Straub

**Affiliations:** Laboratory of Experimental Rheumatology and Neuroendocrine Immunology, University Hospital of Regensburg, D-93053 Regensburg, Germany

## Abstract

Chronic inflammation in rheumatoid arthritis (RA) is accompanied by activation of the sympathetic nervous system, which can support the immune system to perpetuate inflammation. Several animal models of arthritis already demonstrated a profound influence of adrenergic signaling on the course of RA. Peripheral norepinephrine release from sympathetic terminals is controlled by cannabinoid receptor type 1 (CB_1_), which is activated by two major endocannabinoids (ECs), arachidonylethanolamine (anandamide) and 2-arachidonylglycerol. These ECs also modulate function of transient receptor potential channels (TRPs) located on sensory nerve fibers, which are abundant in arthritic synovial tissue. TRPs not only induce the sensation of pain but also support inflammation via secretion of pro-inflammatory neuropeptides. In addition, many cell types in synovial tissue express CB_1_ and TRPs. In this review, we focus on CB_1_ and transient receptor potential vanilloid 1 (TRPV1)-mediated effects on RA since most anti-inflammatory mechanisms induced by cannabinoids are attributed to cannabinoid receptor type 2 (CB_2_) activation. We demonstrate how CB_1_ agonism or antagonism can modulate arthritic disease. The concept of functional antagonism with continuous CB_1_ activation is discussed. Since fatty acid amide hydrolase (FAAH) is a major EC-degrading enzyme, the therapeutic possibility of FAAH inhibition is studied. Finally, the therapeutic potential of ECs is examined since they interact with cannabinoid receptors and TRPs but do not produce central side effects.

## Introduction

Rheumatoid arthritis (RA) is a debilitating disease that affects around 1.3 million people in the US alone [1]. Important characteristics of RA are inflammation of the joint with subsequent destruction of cartilage, pannus formation and infiltrates of immune cells [2–4]. Ongoing inflammation also leads to systemic changes manifesting in co-morbidities like dyslipidemia, depression, fatigue, insulin resistance, activation of the sympathetic nervous system, and cachexia [5, 6]. Changes in sympathetic activity lead to a metabolic switch, which is in part responsible for the perpetuation of inflammation and the increase in cardiovascular risk in RA patients [7].

Cannabis has been used since 4000 BC for the treatment of spasms and post-operative pain [8]. In the 1990s, the two main receptors for cannabinoids (cannabinoid receptors I and II; CB_1_ and CB_2_) were identified [9, 10]. Both receptors are activated by the psychoactive component of cannabis, tetrahydrocannabinol (THC), and several other synthetic and plant-derived cannabinoids [11]. Two major endogenous cannabinoids (endocannabinoids, ECs), arachidonylethanolamine (anandamide, AEA) and 2-arachidonylglycerol (2-AG), were described shortly after the discovery of CB_1_ and CB_2_ [12, 13]. In recent years, other receptors such as transient receptor potential vanilloid 1 (TRPV1), GPR55, or GPR18 were found to bind cannabinoids, and activation of these receptors is responsible for the off-target effects of several cannabinoids [14–18]. Transient receptor potential channel (TRP) modulation by cannabinoids might be explicitly important since these receptors not only influence sensation of pain, but also support inflammation [19].

This review describes physiological aspects of CB_1_ receptors, pharmacological roles of ECs and the EC-degrading enzyme fatty acid amid hydrolase (FAAH), functional crosstalk between ECs and TRPV1, the interaction between ECs and the sympathetic nervous system in RA, the influence of ECs on arthritis disease sequelae in mice and humans, and direct immunomodulatory effects of CB_1_ signaling in the periphery and in the brain. Considering this knowledge we finally try to demonstrate an optimum therapeutic EC approach in RA.

## Physiology

### CB_1_ influences cell function by controlling neurotransmitter levels

**Fig. 1:**
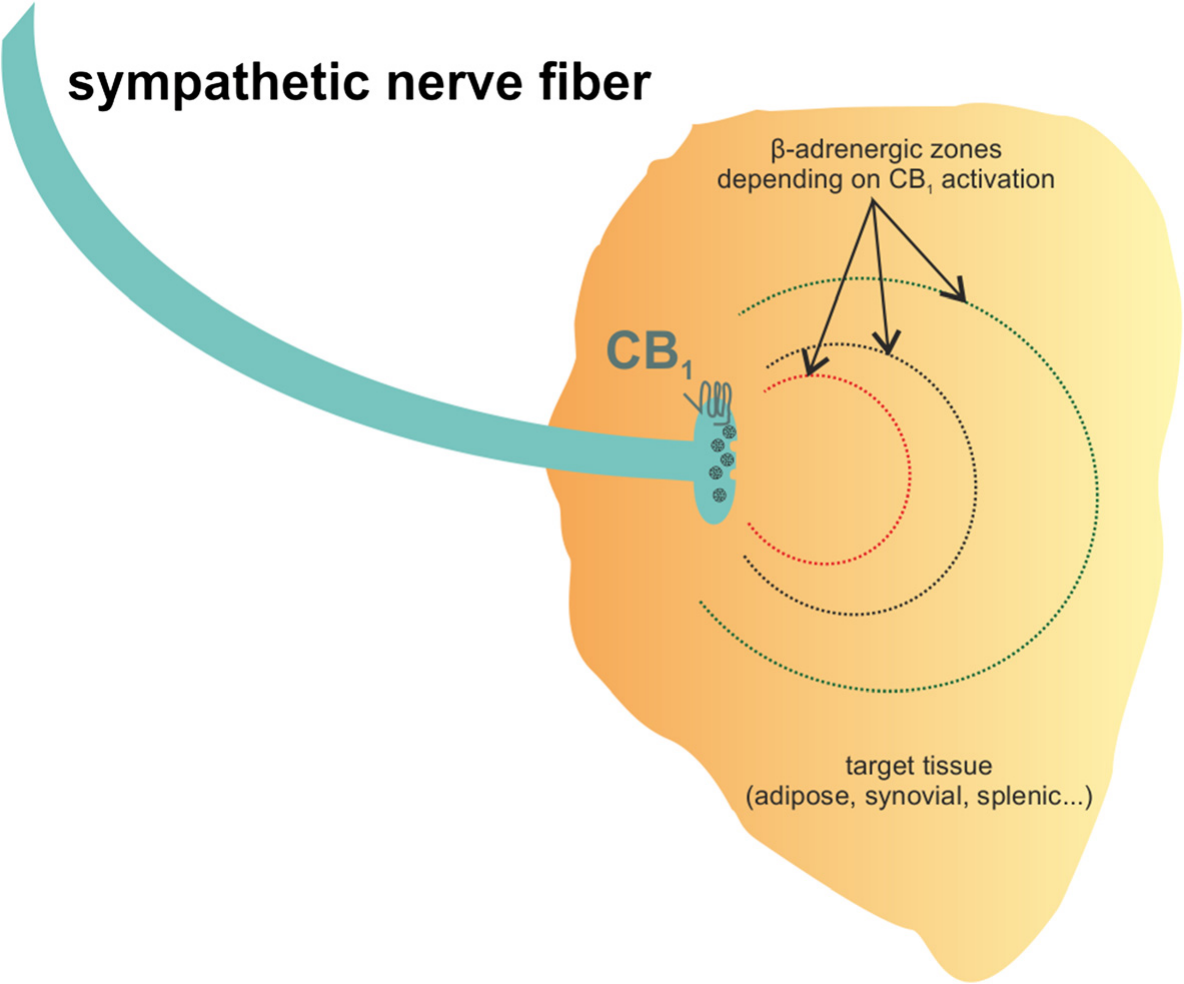
Effects of CB_1_ activation or inhibition on norepinephrine (NE) release in tissue. CB_1_ regulates the amount of NE released from sympathetic nerve terminals. The red zone depicts the effects of CB_1_ agonism, which decreases NE release. Only cells within the red line boundary can be modulated by β-adrenergic receptors under CB_1_ activation. Beyond the dotted 'β-adrenergic zone', α-adrenergic effects prevail. Under basal conditions, the β-adrenergic area is increased (black dotted line). Under CB_1_ inhibition, NE release is boosted and maximal β-adrenergic effects can be achieved (green dotted line). Beta receptor activation on immune cells decreases production of pro-inflammatory mediators, for example, tumor necrosis factor

The classic function of ECs in the nervous system is the regulation of neurotransmitter release via CB_1_, which is also responsible for the psychotropic effects of cannabis [20–23]. CB_1_ is mainly located on presynaptic nerve terminals, and activation of this receptor reduces the release of neurotransmitter from corresponding neurons in a heteroreceptor-typical way [24]. Thus, cannabinoids can increase or decrease neuronal excitability depending on neurotransmitter and brain region affected. CB_1_ receptors are also abundant on peripheral sympathetic nerve terminals, where they modulate adrenergic signaling. This influence on sympathetic nerves can alter lipolysis, cytokine production, ghrelin production, heart rate and bone resorption [20, 25–28]. The effects of CB_1_ activation or inhibition on neurotransmitter release in a given peripheral tissue are depicted in Fig. 1. In addition, CB_1_ receptors are located on nociceptive nerve fibers. Here, CB_1_ agonism increases the threshold for the generation of action potentials via modulation of ion channels and TRPs [29, 30].

Direct effects of CB_1_ activation on immune cells have only been scarcely described. Our group but also others demonstrated an influence of cell adhesion in response to CB_1_ agonism; this effect might also modulate immune function by regulating cell trafficking and tissue extravasation [31, 32].

### CB_2_ regulates immune cell function directly

While CB_1_ functions mainly through modulation of central and peripheral neurotransmitter release, activation of CB_2_ elicits direct anti-inflammatory effects in target cells [33]. This includes reduction of cytokine and matrix metalloproteinase production, modulation of adhesion and migration but also induction of apoptosis [33]. The anti-inflammatory potential of CB_2_ was also confirmed in mouse models of arthritis [34, 35]. While the impact of CB_2_ on immune function has already been investigated and reviewed elsewhere [33, 36], this review focuses on CB_1_.

## Pharmacology

### Role of the ECs anandamide and 2-AG

The action of ECs is limited by rapid degradation involving FAAH, which degrades AEA and related N-acylethanolamines, and monoacylglycerol-lipase (MAGL), which degrades 2-AG [37]. In addition, several enzymes like cyclooxygenase-2, lipoxygenase or cytochrome P450 and others contribute to EC metabolism [38]. Characteristics of AEA, 2-AG, THC and the CB_1_ antagonist rimonabant are given in Table 1. Inhibition of FAAH raises the levels of the N-acylethanolamines AEA, palmitoylethanolamine (PEA) and oleoylethanolamine (OEA) [39]. While AEA is responsible for maintaining basal EC signaling, 2-AG mediates strong and rapid feedback via CB_1_ receptors [40]. This is also reflected by the fact that AEA is a partial agonist at CB_1_, while 2-AG acts as full agonist [41]. Due to its full agonistic properties, elevation of 2-AG by inhibition of MAGL leads to functional antagonism (discussed below) of CB_1_, although this might be prevented by reduced dosing [42, 43]. Furthermore, MAGL inhibition might be detrimental in some situations, since 2-AG is also degraded by cyclooxygenase-2 leading to pro-inflammatory metabolites [44]. Therefore, this review only covers the consequences of FAAH inhibition.

**Table 1:** Characteristics of selected cannabinoid receptor ligands

Ligand	Target receptors	Ki at CB_1_ in nM	Ki at CB_2_ in nM	E_max_/IC_50_ at TRPV1 (nM)	Route of degradation
Anandamide	CB_1_, CB_2_, GPR55, TRPV1, TRPA1, TRPM8 (antagonist)	239.2 ± 61.77 [158]	439.5 ± 95.89 [158]	458 (E_max_) [159]	FAAH, FAAH-2, NAAA, COX-2, LOX [160]
2-AG	CB_1_, CB_2_, TRPV1, GABAA	3423.6 ± 3288.24 [158]	1193.8 ± 327.71 [158]	750 ± 40 (IC_50_) [161]	MAGL, COX-2, LOX, ABHD6/12 [160,162]
Delta9-THC	CB_1_, CB_2_, GPR18	25.1 ± 5.54 [158]	35.2 ± 5.86 [158]	NA	CYP2C [163]
Rimonabant	CB_1_, MOR	1.98 ± 0.36 [164]	NA	NA	CYP3A [165]

Anandamide, 2-arachidonylglycerol (2-AG) and tetrahydrocannabinol (THC) are CB_1_/CB_2_ agonists, rimonabant is a CB_1_/MOR antagonist/inverse agonist. Anandamide and THC are partial CB_1_/CB_2_ agonists, 2-AG is a full agonist at both receptors. The main degrading enzyme for each compound is highlighted in bold. *ABHD*, α/β-hydrolase domain; *CB*_*1*_ */CB*_*2*_, cannabinoid receptor I/II; *COX-2*, cyclooxygenase-2; *CYP*, cytochrome P450; *Delta9 THC*, delta9 tetrahydrocannabinol; *Emax*, maximal functional response; *FAAH*, fatty acid amide hydrolase; *IC50*, half maximal inhibitory concentration; *Ki*, dissociation constant; *LOX*, lipoxygenase; *MAGL*, monoacylglycerol lipase; *MOR*, μ opoid receptor; *NA*, not applicable; *NAAA*, N-acylethanolamine-hydrolyzing acid amidase;* TRPA1*, transient receptor potential ankyrin I; *TRPM8*, transient receptor potential melastatin 8; *TRPV1*, transient receptor potential vanilloid I

### The conundrum of functional antagonism at CB_1_ and TRPV1

Throughout this review, similar effects of CB_1_ agonists and CB_1_ antagonists on features of arthritic inflammation are described. This conundrum can be explained by rapid desensitization and downregulation/internalization of CB_1_ upon agonist exposure [45–47]. If desensitization is disturbed due to mutations in crucial CB_1_ phosphorylation sites, CB_1_ agonism leads to enhanced acute effects and delayed tolerance [48]. Consequently, CB_1_ signaling diminishes in response to repeated agonist exposure [49]. This feature of CB_1_ explains functional antagonism: administration of exogenous cannabinoids or elevation of endogenous levels of the full CB_1_ agonist 2-AG leads to downregulation of CB_1_. If levels drop low enough, production of ECs is not sufficient to activate CB_1_ or CB_1_ signaling pathways. This phenomenon was described with MAGL inhibitors, which increase levels of 2-AG [42]. Another possibility to achieve antagonistic effects with agonists is the use of CB_1_ partial agonists like AEA, which lack full activation of CB_1_ signaling pathways. These partial agonists act as antagonists when full agonists are also present [50].

TRPs, in particular TRPV1, TRPV2, TRPV3, TRPV4, TRPA1 and TRPM8, serve as ionotropic cannabinoid receptors and they also desensitize upon agonist exposure [51–55]. The EC AEA is an agonist at TRPV1 with a binding affinity similar to that of the hot pepper ingredient capsaicin, although it does not activate the receptor like capsaicin [56]. Therefore, although being an agonist itself, AEA prevents the effects of high efficacy agonists like capsaicin, thus serving as antagonist in this setting. Furthermore, AEA rapidly desensitizes TRPV1, which results in reduced calcium influx [57]. In addition, the AEA congeners and FAAH substrates PEA and OEA also desensitize TRPV1 [58, 59]. Although there are no data available regarding the desensitization of other TRPs by N-acylethanolamines, it is likely that this also occurs since there is extensive crosstalk between, for example, TRPV1 and TRPA1 via intracellular calcium [60]. Moreover, it has been demonstrated that synthetic cannabinoid ligands binding TRPA1 also desensitized target cells to the action of TRPV1 agonists [61].

### FAAH inhibition does not produce central side effects and bridges TRPs and cannabinoid receptors

Central activation of CB_1_ has psychotropic side effects and this problem is circumvented by the use of FAAH inhibitors [62]. In contrast to exogenous cannabinoids, AEA does not lead to tolerance at CB_1_ or psychotropic effects [63]. Therapeutically, reduction of tolerance to CB_1_ agonists with FAAH inhibitors can be important since this process leads to a loss of efficacy when repeatedly administered [63]. In addition, elevation of OEA and PEA also provide anti-inflammatory, neuroprotective effects and they enhance neurogenesis mostly via peroxisome-proliferator activated receptors [64–66]. FAAH inhibition has already been demonstrated to be effective in collagen-induced arthritis in mice, although this was attributed to CB_2_ activation [34]. Furthermore, FAAH inhibition not only combines anti-inflammatory effects of several N-acylethanolamines but also targets additional receptors such as TRPV1 and peroxisome proliferator activated receptors [65, 67–69]. One important receptor for AEA and its congeners OEA and PEA is the TRPV1 cation channel, although other TRPs are similarly activated by AEA [69–71].

Besides CB_1_ and CB_2_, ECs as well as synthetic and phytocannabinoids bind to members of the TRP family [54, 61, 72–74]. Several of these non-selective cation channels integrate external and endogenous stimuli and are sensitized and activated during inflammation [19, 75]. Pharmacological elevation of AEA in the rat leads to activation but also desensitization of TRPV1, resulting in increased pain thresholds [69]. In contrast to CB_1_ activation, TRP activation increases cell excitability leading to increased release of neurotransmitters [76–78]. When co-expressed, CB_1_ agonism decreases TRPV1 channel activity by dephosphorylation, which increases the threshold for agonists [78]. Although mainly located on sensory Aδ and C-fibers, TRPs are also expressed on peripheral cells such as synoviocytes, and activation results in increased expression of inflammatory mediators [75, 79, 80]. The best described example of subsequent TRPV1 and CB_1_ activation is the regulation of blood pressure, where only the CB_1_/TRPV1 agonist AEA elicited a triphasic response involving both receptors [81]. First, AEA activates TRPV1 causing hypotension and bradycardia followed by a pressor phase with increased heart rate. In the final phase, prolonged hypotension by AEA is observed and this effect was inhibited by CB_1_ antagonism. The sequential activation of TRPV1 and CB_1_ in the context of blood pressure regulation has been reviewed elsewhere [81].

## Clinical relevance

### The sympathetic nervous system supports chronic inflammation in arthritis - links to endocannabinoids

**Fig. 2:**
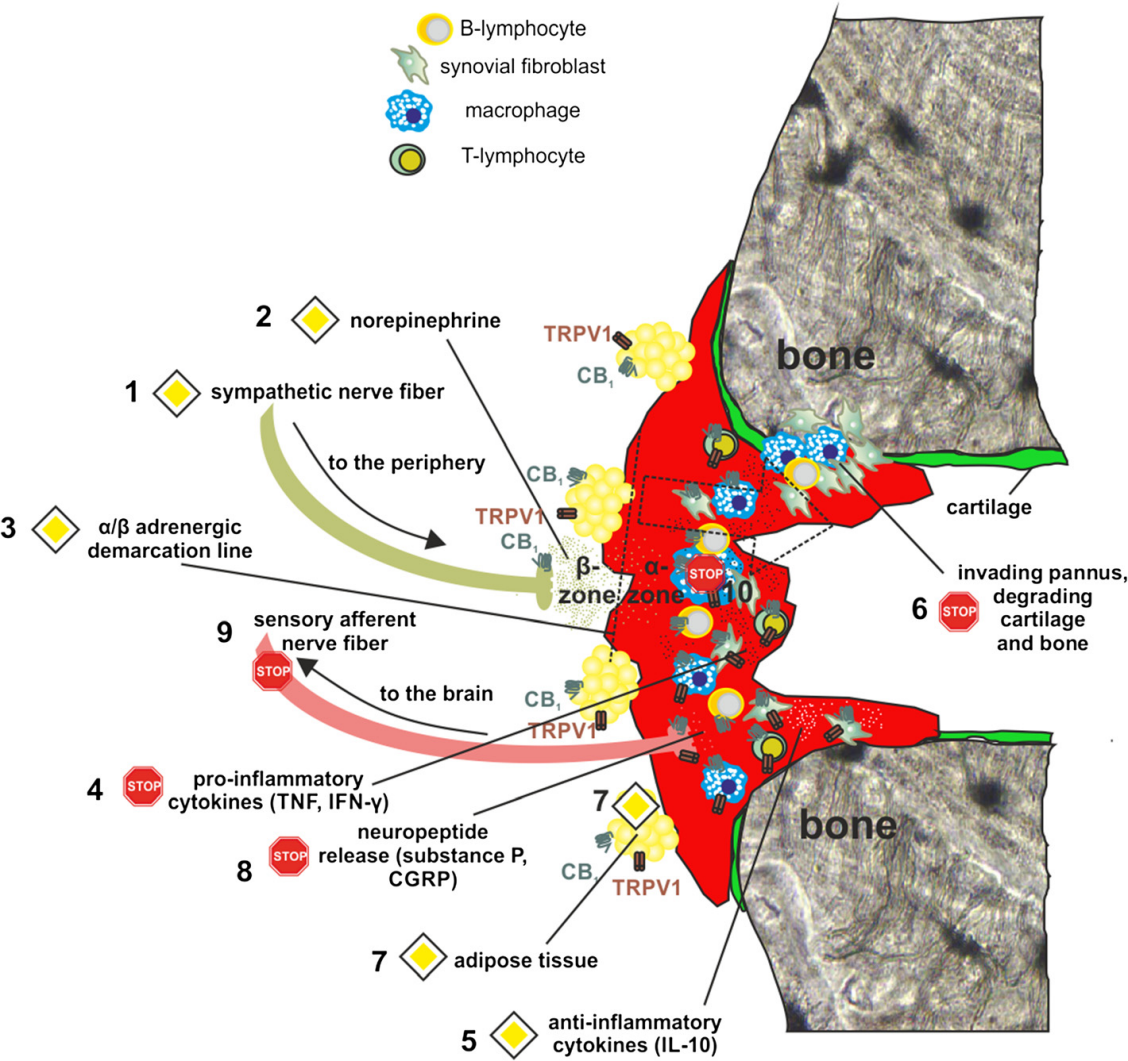
Possible effects of CB_1_ antagonism and fatty acid amid hydrolase (FAAH) inhibition on inflammation in the joint. During the course of arthritis, sympathetic nerve fibers are repelled from synovial tissue (1). Released norepinephrine (NE) (2) stimulates lipolysis, since concentrations are high enough to activate β-adrenergic receptors on adipocytes. Synovial tissue NE concentrations, however, are below the threshold for β-activation. Beyond the 'α/β demarcation line' (3), only pro-inflammatory α-adrenergic signaling is expected. Hypothetically, inflammation can be blocked in the following way. Firstly, CB_1_ antagonism shifts the α/β demarcation line (indicated by dotted arrow) due to enhanced release of NE and its co-transmitters. Secondly, concomitant FAAH inhibition increases local endocannabinoid/N-acylethanolamine concentrations, which enhance sprouting of sympathetic fibers back into synovial tissue. This is followed by a sequence of events: an increase in NE decreases the production of pro-inflammatory cytokines (4) and increases the production of anti-inflammatory cytokines (5). This would reduce cartilage and bone destruction (6). Lipolysis is increased under these conditions since CB_1_ antagonism leads to direct lipolytic effects on adipocytes (7), which are enhanced by β-adrenergic activation. In addition, TRPV1 activated by FAAH inhibition can also contribute to lipolysis (7). Although blockade of CB_1_ enhances TRPV1 sensitization on sensory nerves, this can be counteracted by TRPV1 desensitization through FAAH inhibition but also by reduction of pro-inflammatory cytokines that sensitize TRPV1 (8). Eventually, this can also lead to a reduction of afferent sensory nerve fiber signaling to the central nervous system (9). Direct anti-inflammatory effects of FAAH substrates and CB_1_ antagonists reduce cytokine levels in the joint (10). The STOP symbol indicates inhibition, the PRIORITY ROAD symbol indicates an enhancement of a given effect. CGRP, calcitonin gene-related peptide; IFN, interferon

**Fig. 3:**
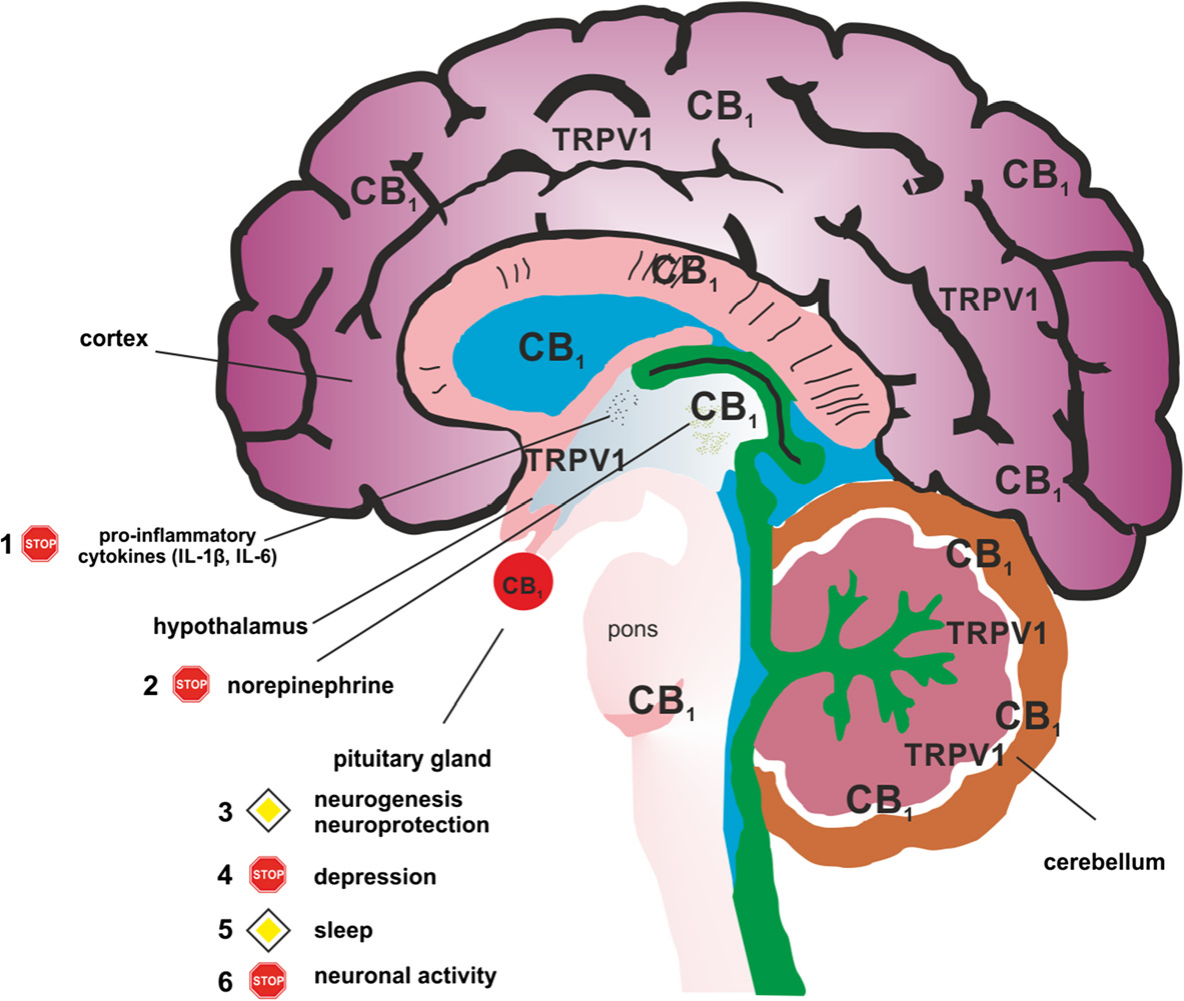
Possible effects of fatty acid amid hydrolase (FAAH) inhibition on neuroinflammation. CB_1_ and TRPV1 are expressed throughout the brain by several cell types, including microglia. In addition, FAAH-degradable N-acylethanolamines activate several other anti-inflammatory pathways supporting the role of CB_1_. Since no data are available regarding the effects of FAAH on sympathetic activity or microglia, the following sequence is hypothetical in nature. Upon activation, microglia produce pro-inflammatory cytokines and CB_1_ activation opposes this (1). Since CB_1_ controls neurotransmitter release, hypothalamic norepinephrine is decreased by FAAH inhibition, restoring brain-immune system-joint communication (2). Damaged neuronal tissue generated by the pro-inflammatory milieu is regenerated by CB_1_ activation (3). FAAH inhibition elevates mood and depressive symptoms in patients disappear due to decreased brain cytokines levels (4). Rheumatoid arthritis patients often suffer from bad sleep quality, and this is surpassed by FAAH inhibition (5). In general, CB_1_ activation decreases neuronal excitability, and this supports the general anti-inflammatory effect on microglia, which are activated by glutamate (6). The STOP symbol indicates inhibition, the PRIORITY ROAD symbol indicates an enhancement of a given effect

Sympathectomy in arthritic patients has already been performed in the 1920s and follow-up studies showed reduced joint swelling and pain in sympathectomized patients [82]. The neuroinflammatory component of arthritis has been revealed in studies by Levine and colleagues [83, 84]. In the mouse model of collagen-induced arthritis it was shown that chemical sympathectomy before or during the time of immunization results in less severe disease [85]. Late sympathectomy, however, results in exacerbation of experimental arthritis, which might be due to deletion of tyrosine hydroxylase-positive catecholamine-producing cells that appear in synovial tissue during the course of the disease [86]. The beneficial effects of tyrosine hydroxylase-positive cells on the development of collagen-induced arthritis was demonstrated by our group. *In vitro*, tyrosine hydroxylase controls cytokine production in mixed synovial cells, whereas *in vivo* introduction of these cells into arthritic mice reduced arthritic score [87]. During arthritic inflammation in mice and humans, production of nerve repulsion factors by macrophages leads to the retraction of sympathetic but not sensory fibers from synovial tissue [88]. As a result, synovial concentration of norepinephrine falls under the threshold for anti-inflammatory β2 receptor activation and this favors pro-inflammatory effects via α-adrenergic signaling [89, 90]. However, sympathetic signaling is increased in adipose tissue surrounding the synovium, which is responsible for generating energy-rich substrates to support inflammation [91]. These changes in sympathetic activity during the course of arthritis might be limited or even reversed by altering either EC production or CB_1_ function, since this receptor controls norepinephrine release. Reduction of EC production by blocking appropriate synthesizing enzymes leads to a functional loss of CB_1_ since low levels of ECs can no longer activate the receptor. This was already demonstrated in a mouse model of constipation, where inhibition of diacylglycerol lipase α lowered levels of the CB_1_ agonist 2-AG with concomitant increases in gut motility [92]. The same effect is achieved by antagonizing CB_1_ directly [93]. The loss of sympathetic nerves, altered adrenergic signaling and the possible influence of ECs in the joint is visualized in Fig. 2. In parallel with the disappearance of sympathetic nerve fibers in the joint, hypothalamic norepinephrine, interleukin (IL)-6 and IL-1β increase during the induction phase of experimental arthritis [94] (Fig. 3). In addition, these changes in cytokine levels and disruption of adrenergic signaling are not accompanied by an adequate response of the hypothalamus-pituitary-adrenal (HPA) axis, resulting in low cortisol levels in relation to inflammation in humans and rodents [94]. A more detailed description of the influence of the sympathetic nervous system on inflammation has recently been published by our group [95].

### Modulation of adrenergic signaling via CB_1_ might be beneficial in arthritis

**Fig. 4:**
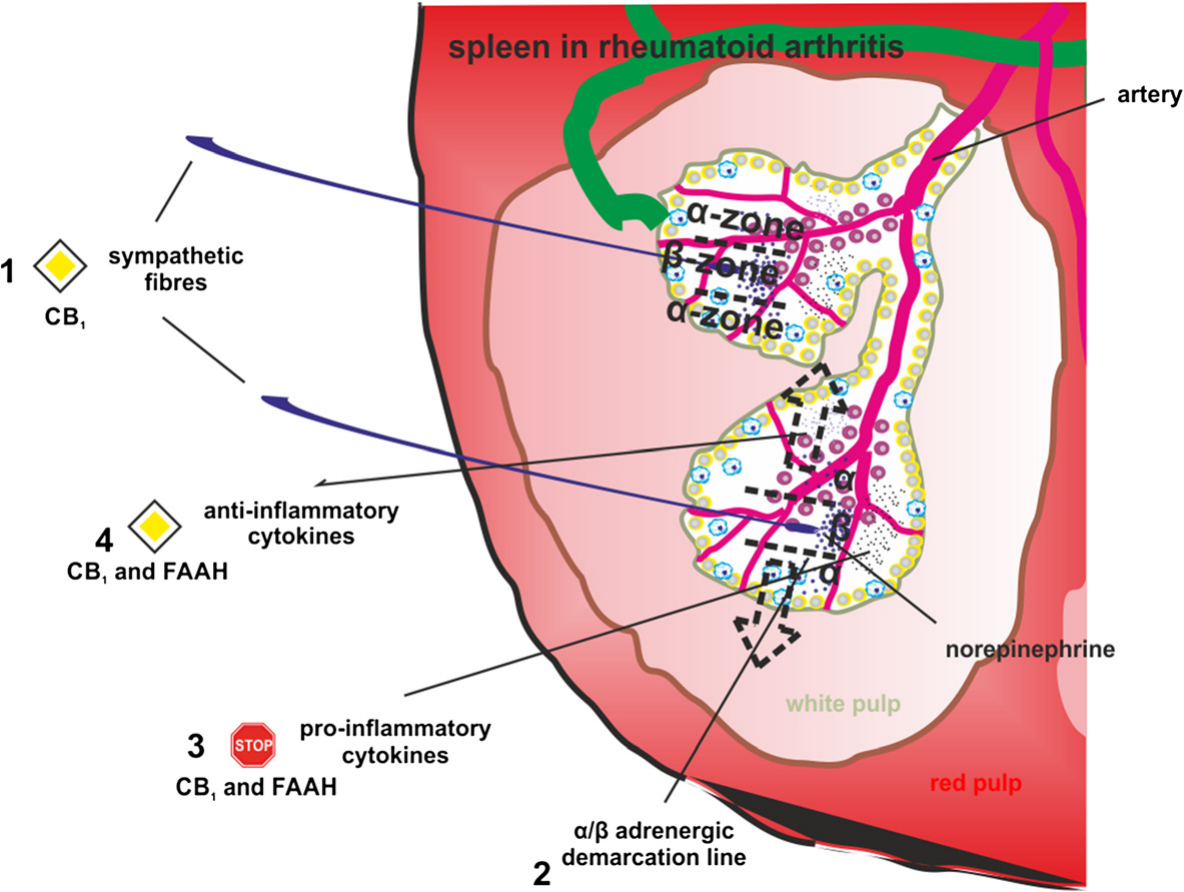
Possible effects of CB_1_ antagonism and fatty acid amid hydrolase (FAAH) inhibition on spleen. The healthy spleen is sympathetically innervated and β-adrenergic signaling prevails. Arthritis leads to a loss of sympathetic fibers and β-adrenergic signaling is decreased in favor of pro-inflammatory α-adrenergic signaling (1). The different signaling zones are depicted by dotted lines. Hypothetically, CB_1_ antagonism leads to increased secretion of norepinephrine and its co-transmitters from sympathetic terminals. While the β-adrenergic zone would be increased (indicated by dotted arrows) (2), pro-inflammatory cytokine production (3) can be decreased with a concomitant rise in anti-inflammatory cytokines (4). Anti-inflammatory effects of β-adrenergic signaling are supported by direct effects of CB_1_ antagonists on immune cells and FAAH substrates engaging TRPV1 and possibly other anti-inflammatory receptors. The STOP symbol indicates inhibition, the PRIORITY ROAD symbol indicates an enhancement of a given effect. The involved mechanism (CB_1_ or FAAH) is given below the symbols

In adjuvant arthritis, immune cells respond to adrenergic β2 receptor stimulation with decreased production of tumor necrosis factor (TNF), an increase in anti-inflammatory IL-10, and a shift to a T-helper type 2 and T-regulatory immune response [96]. Antagonism of CB_1_ at splenic sympathetic terminals provides strong anti-inflammatory effects and ameliorates collagen-induced arthritis in mice via reduction of TNF levels, which was inhibited by β2 adrenergic antagonism [26] (Fig. 4). Furthermore, β2 adrenergic activation on murine B-lymphocytes increases production of the anti-inflammatory cytokine IL-10, which inhibits inflammation [97]. The time window for anti-arthritic β2 adrenergic effects in mice is crucial since early activation (during the induction phase of experimental arthritis) of sympathetic signaling in the spleen increases interferon (IFN)-γ production [98]. Sympathetic innervation of the spleen is reduced during the course of experimental arthritis, comparable to the situation in synovial tissue [99]. This has profound effects on local adrenergic signaling since low concentrations of norepinephrine favor pro-inflammatory α-adrenergic receptor activation [100, 101] (Fig. 4). Although the beneficial outcome of CB_1_ receptor antagonism in collagen-induced arthritis in mice was attributed to β2-receptor activation on splenocytes, several other mechanisms might contribute to the therapeutic effects. CB_1_ antagonism at sympathetic terminals surrounding the synovium might have different outcomes depending on the magnitude of recovery of norepinephrine levels in the joint. If β2 signaling is restored in synovial tissue, local concentrations of IFN-γ and TNF might decline, leading to an overall decrease in joint destruction, synovial inflammation and pain [102, 103] (Fig. 2). On the other hand, since we demonstrated an increase of sympathetic fibers in human synovial adipose tissue, increased norepinephrine release might further increase lipolysis and thereby fuel inflammation [91]. Thus, it is imperative to maintain norepinephrine levels over a certain 'β2 activation threshold' in the synovium, which might only be achieved with continuous high doses of CB_1_ antagonists. Consequences of enhanced β2 signaling by CB_1_ antagonism are depicted in Fig. 2.

Although the above mentioned stimulating effects of CB_1_ antagonism on adrenergic signaling are evident, CB_1_ agonists might also prove useful in modulating arthritis. As mentioned earlier, sympathectomy in the early phase ameliorates experimental arthritis in mice [85]. This indicates a pro-inflammatory influence of adrenergic signaling at the beginning of the disease, which might be counteracted by CB_1_ agonists decreasing norepinephrine levels [20]. Arthritis is accompanied by a loss of sympathetic nerve fibers from sites of inflammation and this might also be counteracted by CB_1_ activation, since neurogenesis is disturbed in CB_1_ knock-out mice, although we do not know whether this also applies for sympathetic nerve fibers [104].

The development of comorbidities such as bone resorption, depression and water retention/volume expansion in RA is partly driven by changes in sympathetic activity [19, 105]. Osteoporosis is a major contributor to RA-associated complications and osteoclasts and osteoblasts respond to cannabinoid receptor activation [106, 107]. Activation of CB_1_ results in enhanced osteoblast differentiation, which leads to reduced osteoporosis. Blockade of CB_1_ disturbs osteoclast function and increases bone mass in the young, but leads to osteoporosis later on due to decreased bone formation [108].

One major disability associated with RA is the development of depression, which affects around 17 % of patients and is associated with poorer disease outcome [109]. Depression and CB_1_ are connected since side effects of rimonabant, a first generation CB_1_ inverse agonist/antagonist, include depression and anxiety while CB_1_ agonism has anxiolytic-like and antidepressant-like activities [110, 111]. The effects of CB_1_ agonism by FAAH inhibition in the brain are depicted in Fig. 3.

Overactivity of the sympathetic nervous system in RA also leads to water retention via activation of the renin-aldosterone-angiotensin system [19]. Cannabinoids not only induce diuretic effects but also decrease aldosterone secretion from the adrenal glands by activation of CB_1_ [112, 113].

### CB_1_ antagonism activates the HPA axis and reverses insulin resistance

Although modulation of immune cell function via β2-adrenergic receptors is important, CB_1_ antagonism also supports beneficial systemic changes. One of the hallmarks of RA is an inadequate cortisol secretion in relation to inflammation [114]. Antagonism at CB_1_ might counteract this phenomenon, since CB_1_ knock-out mice had higher levels of adrenocorticotropic hormone and corticosterone under basal but also under stressed conditions [115]. ECs control glucocorticoid feedback and, therefore, CB_1_ antagonism increases circulating adrenocorticotropic hormone levels [116]. Interestingly, high doses of a CB_1_ agonist also increase the activity of the HPA axis, although this is due to alteration of serotonergic and adrenergic transmission [117]. The same outcome using CB_1_ antagonism or agonism on HPA axis activation might also depend on the concentration of CB_1_ agonists and whether central or peripheral CB_1_ receptors are targeted. Peripheral agonism at CB_1_ leads to subsequent activation of α and β adrenoreceptors, which are linked to the antinociceptive effects of CB_1_ in a rat pain model [118]. Increases in adrenergic signaling by CB_1_ agonists might be due to decreased inhibitory gamma-aminobutyric acid (GABA) signaling since release of this neurotransmitter is also controlled by CB_1_ [22]. Thus, enhanced GABA signaling reduces sympathetic activity and vice versa [119]. Central activation of CB_1_ mediates the rapid effect of glucocorticoid negative feedback and this might explain the necessity for high peripheral doses of the CB_1_ antagonist rimonabant to increase cortisol levels [120, 121].

A major problem during the course of RA is the development of insulin resistance with systemic metabolic changes [122, 123]. Insulin resistance is a direct consequence of enhanced pro-inflammatory cytokine signaling and TNF, IL-6, IL-1β as well as other cytokines are responsible for these changes [124]. From 2006 to 2008 the CB_1_ antagonist rimonabant was marketed for use against obesity but was withdrawn due to central side effects [125]. However, the drug proved to be effective at decreasing important parameters associated with metabolic syndrome. Rimonabant reduces leptin expression, decreases atherosclerosis, and reverses insulin resistance in rodents and humans [126, 127]. In this respect, CB_1_ antagonism might also be beneficial in reversing metabolic changes in RA. Insulin resistance is induced by the immune system to divert energy to active immune cells, which are not dependent on insulin for glucose utilization [105]. Therefore, CB_1_ antagonism might normalize energy distribution throughout the body and this might deprive the activated immune system of nutrients important for the perpetuation of inflammation. Interestingly, CB_1_ activation by the phytocannabinoid δ9-tetrahydrocannabinol (THC) corrects hyperlipidemia and hyperglycemia [128]. This effect might be relevant when using CB_1_ partial agonists like THC that act as antagonists when full agonists like the EC 2-AG are present. In mice, THC antagonized the effects of the synthetic CB_1_ agonist AM2389 on hypothermia, although it elicited hypothermic effects by itself [129]. Furthermore, repeated administration of cannabinoids leads to desensitization and downregulation of CB_1_, resulting in functional antagonism [42, 48].

Systemic leptin levels are decreased by CB_1_ inhibition, and this adipokine is associated with higher IL-6 production and it also initiates production of TH1 cytokines [130, 131]. In addition, cardiovascular events are a major risk in RA and CB_1_ antagonists might be effective in decreasing vascular inflammation [132, 133].

### Direct anti-inflammatory effects of CB_1_ on immune cells

Although most changes associated with CB_1_ antagonism are mediated via the sympathetic nervous system, direct effects on the immune system are also described. In macrophages from CB_1_ knock-out mice, TLR4 expression and concomitant pro-inflammatory cytokine production were reduced [134]. Anti-inflammatory effects of CB_1_ inhibition were also demonstrated in THP-1 macrophages, where rimonabant decreased TNF and increased IL-10 production [135]. Furthermore, in a mouse model of sponge-induced angiogenesis, CB_1_ antagonism reduced leukocyte infiltration and chemokine/cytokine production [136].

CB_1_ agonism also has anti-inflammatory effects on immune cells - for example, decreased activation of T lymphocytes by downregulating IL-2 [137]. However, direct effects of CB_1_ agonists are most prominent when injected into the brain, where CB_1_ activation reduces the severity of intestinal inflammation and decreases the activity of microglial cells via reduction of pro-inflammatory cytokines in mice [138, 139]. Therefore, CB_1_ activation might alleviate arthritis through central nervous pathways, since neuroinflammation and concomitant increases in brain cytokine levels contribute to the disease [94, 140].

### Central effects of CB_1_ ligands limit their therapeutic use

Although therapeutically active when administered intrathecally, the use of CB_1_ agonists or antagonists is limited due to their central adverse events. While CB_1_ antagonists/inverse agonists like rimonabant induce depression and anxiety in some patients, CB_1_ agonists like THC have psychotropic properties [110, 141]. This might derive from reduction of glutamatergic neurotransmission in response to CB_1_ activation leading to effects similar to NMDA antagonism [142]. This problem might be circumvented by using peripherally restricted CB_1_ ligands, which have been generated as second generation cannabinoid therapeutics with proven effects [143, 144]. Furthermore, neutral antagonists with limited brain penetration and which lack the adverse effects of the inverse agonist rimonabant have been developed [145]. Neutral antagonists do not influence the constitutive activity of CB_1_ and therefore do not mediate some of the adverse effects observed with rimonabant therapy [146]. In contrast to neutral antagonists, inverse agonists like rimonabant not only block CB_1_ but also stabilize the receptor in an inactive conformation. This diminishes basal signaling and leads to a reciprocal receptor response. In the case of CB_1_, cAMP is increased by inverse but not by neutral antagonists [146, 147].

### Crosstalk between CB_1_ and TRPV1 modulates pain and inflammation in arthritis

The importance of TRPV1 in arthritis is emphasized in knock-out animals that show an attenuated disease [148, 149]. In TRPV1^−/−^ animals, pain thresholds were increased with a concomitant reduction of joint inflammation [149]. The same protective effect was achieved by oral administration of the TRPV1 agonist SA13353, which reduced TNF production and provided anti-arthritic effects in the rat [150]. Interestingly, this effect was mediated by TRPV1 located on sensory neurons, emphasizing the neuronal component of arthritis [150]. This might disrupt a positive feedback loop, since TNF and other pro-inflammatory cytokines sensitize TRPV1 and enhance its activity [102]. The paradoxical finding that TRPV1 agonists also act in an anti-inflammatory fashion is explained by rapid desensitization of TRPV1 in response to agonist treatment, which depends on the agonist used [151]. Findings in synovial fibroblasts support this notion, where the TRPV1 agonist capsaicin increases IL-6 production, while AEA, a low efficacy TRPV1 agonist, decreased IL-6 levels under TNF stimulation (T Lowin, unpublished data) [80].

Since some peripheral effects of TRPV1 are attributed to receptors located on sensory nerve terminals which co-express CB_1_, crosstalk between both receptors might define the outcome of inflammation [152]. This can be important in RA, since elevated synovial levels of nerve growth factor sensitize TRPV1 to inflammatory stimuli and CB_1_ agonism counteracts this response [153, 154]. In this respect, FAAH inhibition might be superior to selective CB_1_ agonists since AEA or its metabolites not only activate CB_1_ but also desensitize TRPV1, leading to analgesia [69]. Neuronal TRPV1 increases neurotransmitter and pro-inflammatory neuropeptide release via elevation of intracellular calcium levels and the same mechanism often induces the secretion of cytokines from immune cells [155–157]. Inhibition of TRPV1 function by concomitant CB_1_ activation and AEA-induced desensitization (FAAH inhibition) might be a promising strategy to reduce RA disease activity and pain.

## Conclusion: is there a perfect cannabinoid-based therapy for the treatment of RA?

The question arises how to modulate the EC system for the treatment of RA. The best treatment option might be a combination of a peripherally restricted CB_1_ antagonist and a FAAH inhibitor raising systemic levels of N-acylethanolamines. CB_1_ antagonism has already been shown to result in anti-arthritic effects in mice and this treatment might also increase adrenergic signaling in RA, thereby reducing TNF and IFN-γ and decreasing joint inflammation and cartilage destruction. Potential effects of CB_1_ antagonism (also of FAAH inhibition) in arthritic synovium and spleen are shown in Figs. 1 and 3, respectively.

Furthermore, CB_1_ antagonists might reverse metabolic alterations associated with RA: for example, insulin resistance, enhanced leptin expression, depression/fatigue or atherosclerosis. FAAH inhibition on the other hand can counteract the neuroinflammatory component of RA by activating neuronal CB_1_ and TRPV1 (Fig. 3). Furthermore, the FAAH substrates OEA and PEA can support anti-inflammatory and neurogenic effects of central CB_1_ activation via peroxisome-proliferator activated receptors. In addition, CB_1_ activation in the brain lowers sympathetic activity, which can decrease disease-related problems like hypertension. In addition, increases in brain AEA can have antidepressant effects and since many RA patients suffer from mood disorders, FAAH inhibition might help to counteract this central nervous system problem.

In the periphery, FAAH inhibition leads to analgesic and anti-inflammatory effects via desensitization of TRPV1. Moreover, FAAH inhibition has been shown to have high efficacy in arthritic mice through activation of CB_2_, which might also be beneficial in patients by downregulating cytokine production. In summary, therapeutic intervention in RA with a peripherally restricted CB_1_ antagonist and a FAAH inhibitor might offer a promising strategy to ameliorate RA.

## Abbreviations

2-AG: 2-arachidonylglycerol
AEA: Arachidonylethanolamine
EC: Endocannabinoid
FAAH: Fatty acid amid hydrolase
GABA: Gamma-aminobutyric acid
HPA: Hypothalamus-pituitary-adrenal
IFN: Interferon
IL: Interleukin
MAGL: Monoacylglycerol-lipase
OEA: Oleoylethanolamine
PEA: Palmitoylethanolamine
RA: Rheumatoid arthritis
THC: Tetrahydrocannabinol
TNF: Tumor necrosis factor
TRP: Transient receptor potential channel
